# Identification of *ALK* Gene Alterations in Urothelial Carcinoma

**DOI:** 10.1371/journal.pone.0103325

**Published:** 2014-08-01

**Authors:** Joaquim Bellmunt, Shamini Selvarajah, Scott Rodig, Marta Salido, Silvia de Muga, Irmgard Costa, Beatriz Bellosillo, Lillian Werner, Stephanie Mullane, André P. Fay, Robert O'Brien, Jordi Barretina, André E. Minoche, Sabina Signoretti, Clara Montagut, Heinz Himmelbauer, David M. Berman, Philip Kantoff, Toni K. Choueiri, Jonathan E. Rosenberg

**Affiliations:** 1 Bladder Cancer Center, Dana-Farber Cancer Institute/Harvard Medical School, Boston, Massachusetts, United States of America; 2 Hospital del Mar Research Institute-IMIM, Barcelona, Spain; 3 Department of Pathology, Hospital del Mar Research Institute-IMIM, Barcelona, Spain; 4 Hospital Parc Tauli, Sabadell, Spain; 5 Biostatistics and Computational Biology, Harvard Medical School, Dana-Farber Cancer Institute, Boston, Massachusetts, United States of America; 6 Broad Institute, Cambridge, Massachusetts, United States of America; 7 Max Planck Institute for Molecular Genetics, Berlin, Germany; 8 Centre for Genomic Regulation (CRG), Barcelona, Spain; 9 Universitat Pompeu Fabra, Barcelona, Spain; 10 Department of Pathology, Johns Hopkins University, Baltimore, Maryland, United States of America; 11 Department of Medicine, Memorial Sloan-Kettering Cancer Center, New York, New York, United States of America; Istituto dei tumori Fondazione Pascale, Italy

## Abstract

**Background:**

Anaplastic lymphoma kinase (*ALK*) genomic alterations have emerged as a potent predictor of benefit from treatment with *ALK* inhibitors in several cancers. Currently, there is no information about *ALK* gene alterations in urothelial carcinoma (UC) and its correlation with clinical or pathologic features and outcome.

**Methods:**

Samples from patients with advanced UC and correlative clinical data were collected. Genomic imbalances were investigated by array comparative genomic hybridization (aCGH). *ALK* gene status was evaluated by fluorescence *in situ* hybridization (FISH). *ALK* expression was assessed by immunohistochemistry (IHC) and high-throughput mutation analysis with Oncomap 3 platform. Next generation sequencing was performed using Illumina Genome Analyzer IIx, and Illumina HiSeq 2000 in the FISH positive case.

**Results:**

70 of 96 patients had tissue available for all the tests performed. Arm level copy number gains at chromosome 2 were identified in 17 (24%) patients. Minor copy number alterations (CNAs) in the proximity of *ALK* locus were found in 3 patients by aCGH. By FISH analysis, one of these samples had a deletion of the *5′ALK*. Whole genome next generation sequencing was inconclusive to confirm the deletion at the level of the *ALK* gene at the coverage level used. We did not observe an association between *ALK* CNA and overall survival, ECOG PS, or development of visceral disease.

**Conclusions:**

*ALK* genomic alterations are rare and probably without prognostic implications in UC. The potential for testing *ALK* inhibitors in UC merits further investigation but might be restricted to the identification of an enriched population.

## Introduction

Urothelial carcinoma (UC) accounts for 15,210 cancer deaths per year in the United States [1]. Five-year survival for patients with muscle invasive (T2) disease or greater is only 50%.

Advanced UC of the bladder is often associated with mutations and multiple somatic copy number alterations [2]. Comparative genomic hybridization studies of bladder carcinomas and cell lines have revealed a number of recurrent genetic aberrations including amplifications or gains on 8q22-24, 11q13, 17q21, and losses on chromosomes 9, 8p22-23, and 17p6-9 [3], [4]. In several clinical cohorts, some of these genomic alterations have also been associated with pathological stage and outcome [5].

In the recent years, potential new targets for treatment intervention have been described in urothelial tumors. The identification of driving genomic alterations as mutations even if occurring in only a small subset of bladder cancer patients, may lead to the development of patient-specific therapies as has been the case of the recently described mutations in *TSC1* predicting response to mTOR inhibitors like everolimus [6]–[8]. Another example is the *PIK3CA* gene, mutated in up to 26% of cases in the series by Ross and colleagues that may predict sensitivity to *PIK3CA*/mTOR inhibitors [9].

The ALK (anaplastic lymphoma Kinase) inhibitor crizotinib, has recently shown high efficacy in the treatment of patients with non-small cell lung cancer (NSCLC) with *ALK* translocation which is present in about 4–7% of the tumors [10]–[12]. In a phase I study of NSCLC patients with an *ALK* translocation, the response rate was 57% independent of performance status or number of previous treatments with a 70% probability of progression free survival at 6 months [13]. In several other tumor types besides lung cancer, *ALK* genomic alterations have been identified as potential oncogenic drivers, meaning that cancers in different organs can be targeted for treatment with *ALK* inhibitors regardless of their cell of origin.

In UC, *ALK* copy number gain, amplification, translocations, mutations, or expression have not been characterized. We therefore investigated *ALK* protein expression and underlying genetic aberrations in a cohort of patients who received chemotherapy in the setting of metastatic disease, focusing on clinical and prognostic implications.

In the present study we show that *ALK* genomic alterations, such as copy number alterations (CNA) and deletions, occur in UC. Additionally, we attempted to identify the impact of these alterations with clinical and outcome features.

## Material and Methods

### Patients

This project was approved by the local ethics committee (CEIC-IMAS) at Hospital del Mar, and by the Dana-Farber/Harvard Cancer Center (DF/HCC) institutional review board (IRB). Because the majority of patients were died at the time of collecting samples, a waiver of consent was requested and given from IRB of DF/HCC for all participants (requiring complete deidentification of the samples prior the analysis).

A cohort of 96 patients, with metastatic UC treated with platinum-based combination was identified. All patients underwent several treatment regimens, all containing gemcitabine and a platinum compound, with some patients receiving additional paclitaxel as well. Patient clinical data was collected. The final cohort included 70 patients (52 males, 18 females) with available clinical data and sufficient tissue samples to conduct all the genomic studies.

### Tumor Samples

The analysis was performed in formalin-fixed paraffin embedded (FFPE) tissue from UC of the urinary tract. Other molecular studies have been performed and reported in these samples in order to characterize the biology of UC [14]. The specimens were retrospectively retrieved from the pathology archive at Hospital del Mar and Mar Biobank in Barcelona, Spain. Slides were reviewed separately by two genitourinary specialist pathologists (MS, DB). All patients had high grade transitional cell carcinoma and no other histological variant was included in this study. Tumor areas were evaluated by a single pathologist (DB) and tumor bearing 0.6 mm cores were punched for DNA extraction and/or tissue microarray (TMA) construction.

### *ALK* analysis

*ALK* genomic alterations were evaluated by array comparative genomic hibridization (aCGH), fluorescence *in situ* hybridization (FISH), immunohistochemistry (IHC), mass spectrometry mutation analysis and next-generation sequencing. Description of methods can be found in the appendix (**Methods S1**).

### Statistical analysis

Statistical analysis of clinical data and molecular features was carried out with SAS version 9.2 (SAS Institute Inc, Cary, NC). Patient and clinical characteristics were summarized as number and percentages for categorical variables and median and inter-quartile ranges for continuous variables. Overall survival (OS) was defined from the date patients received first line chemotherapy for advanced disease until date of death or censored on the last known alive date. *ALK* copy number alteration was defined as having more than a 4 fold change [15]. Fisher exact test was used to assess the associations of *ALK* copy number alteration with ECOG PS and whether patients developed visceral disease. Cox proportional hazard model was used to assess the associations of *ALK* copy number alteration and overall survival in both univariate and multivariate analyses. Kaplan-Meier estimate was used to summarize median overall survival. All the statistical tests were conducted at the two-sided 0.05 level of significance.

## Results

The median OS was 12 months with 45 patients deceased at the time of analysis, with a median follow-up of 23 months. **Table 1** summarizes patient and clinical characteristic for the entire cohort as well as for patients with more than 4 fold copy number gain in the FISH analysis.

**Table 1:** Patients and Clinical Characteristics.

	All patients (N = 70)		Patients with copy number alteration (N = 17)	
	N	% or median (q1, q3)	N	% or median (q1, q3)
**Age**	61	63 (54, 68)	15	66 (58, 68)
**Sex**				
Male	52	74%	15	88%
Female	18	16%	2	12%
**ECOG PS**				
0	22	31%	4	24%
1, 2	48	69%	13	76%
**Visceral diseases**				
No	41	59%	7	41%
Yes	29	41%	10	59%
**Pathological stage**				
Stage 0 (Ta)	5	7%	2	12%
Stage I (T1)	5	7%	0	0%
Stage II (T2)	36	51%	8	47%
Stage III (T3, T4)	22	31%	7	41%
Stage IV (L, M)	1	1%	0	0%
Missing	1	1%	0	0%

### Recurrent chromosomal gains and losses by aCGH

Analysis by aCGH of the 70 patients included in the study identified 95 focal and 21 broad (identified as >50% of the chromosome arm) events. The results of the broad alteration analysis were largely consistent with the current literature [16]–[18]. We observed frequent losses of chromosomes 5q (43%), 8p (69%), 9 (p: 48%; q: 41%), 10q (41%), 11p (49%), 17p (51%), and 22q (40%) and recurrent gains of chromosomes 3q (46%), 5p (48%), 8q (48%), 19q (34%), and 20 (60%). Three specimens out of 70 harbored minor non-significant alterations (log2 ratio 0–0.8) in chromosome 2, where *ALK* gene locus is located. This encouraged us to conduct a more in-depth search of *ALK* genomic alterations and to further characterize the 5′*ALK* deletion seen by FISH in one patient.

### FISH analysis of *ALK* gene/copy number gains

**Figure 1:**
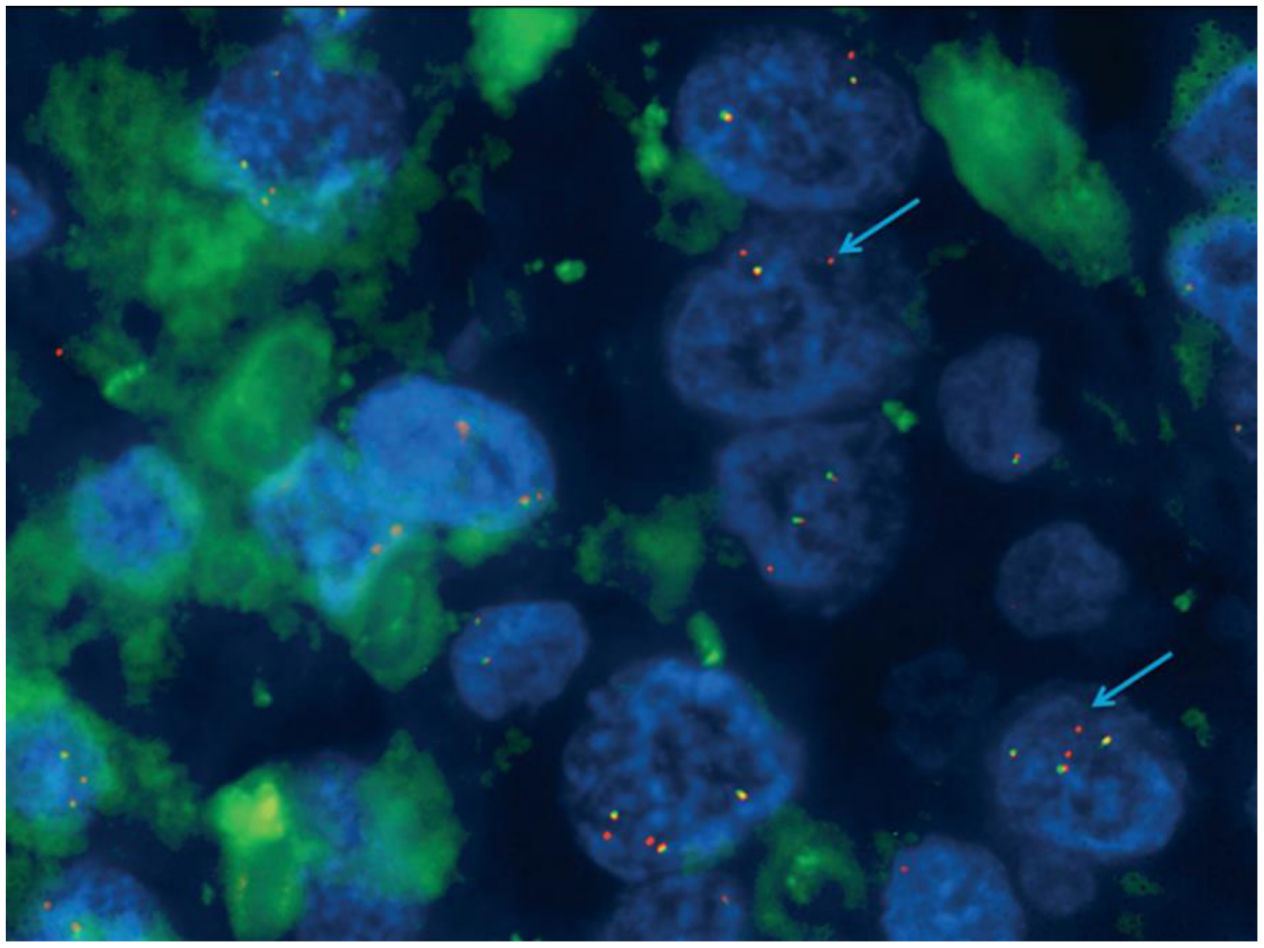
1298case –FISH + for ALK variant (green probe missing).

To further characterize genomic imbalances on chromosome 2, all samples underwent FISH analysis. One case presented a deletion of the green signal (5′*ALK*), centromeric to the *ALK* gene, and also had gain of the *ALK* gene fusion signals and 3′*ALK* signal (**Figures 1** **and** **2**). This FISH pattern was interpreted as an *ALK* atypical rearrangement as has been described in *ALK* positive NSCLC because a single orange (3′*ALK*) signal was seen [19]. In these cases it is assumed that the deletion is the result of translocation. Analyses of EML4 as well as other known fusion partners such as TGF and KIF5 were performed without finding any translocation of these genes. Even so, it is possible that the deletion does not cause the *ALK* translocation and other molecular techniques need to be applied to further characterize the FISH findings.

**Figure 2:**
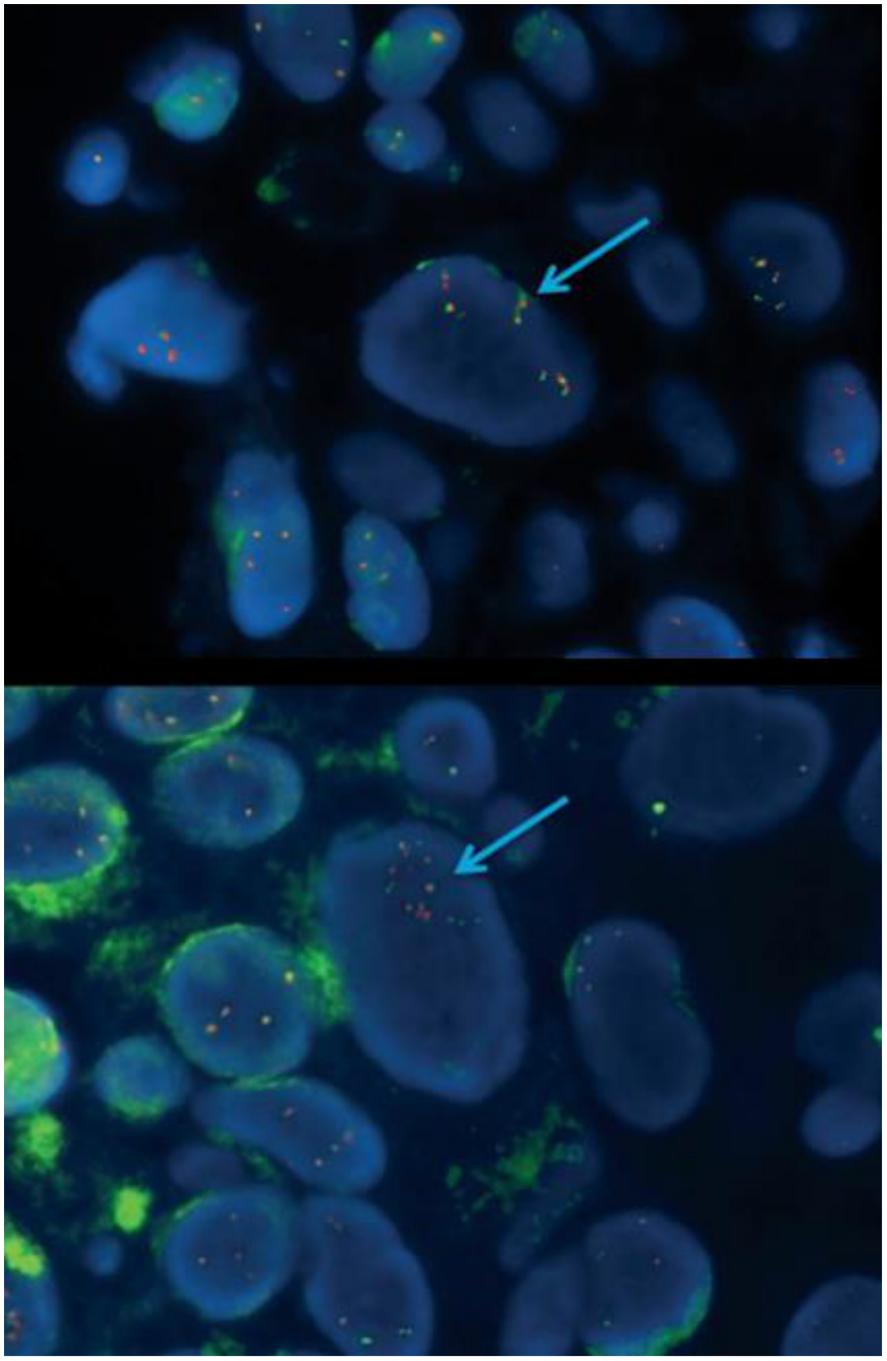
1298case –FISH copy gain (a) & amplified (b).

**Figure 3:**
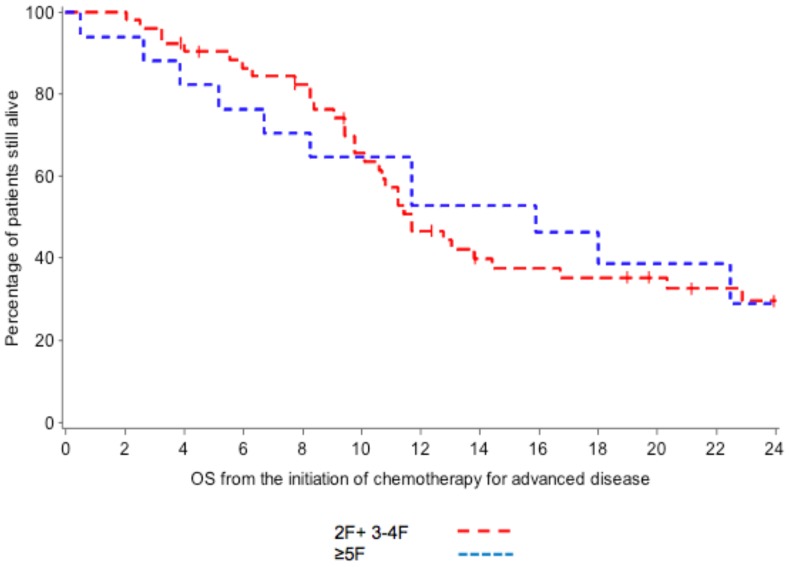
Comparison of OS between ≥5F patients and 2F+3−4F.

*ALK* gene copy number gains and amplification were analyzed in all samples. Two cases presented amplification of *ALK*. 90% of samples showed *ALK* copy number gain due to polysomy of chromosome 2. All of them had 3 to 6 copies of CEP2 except one case with high polysomy. Among 70 urothelial tumors, 7 (10%) demonstrated 2F signals (2 intact *ALK* loci), 46 (65.7%) had 3–4F signals present, and 17 (24.3%) had ≥5F signals (range 5F–11F; median 6F) in >10% of nuclei (**Table 2**). The associations of *ALK* copy number alteration with ECOG PS, visceral disease, and OS are summarized in **Tables 3** **and** **4**. No significant association between *ALK* copy number alteration and clinical features or overall survival was observed (**Figure 3**).

**Table 2:** Copy Number Alteration.

	N	%
**2F**	7	10
**3-4F**	46	66
**≥5F**	17	24

**Table 3:** Association of *ALK* copy number alteration with ECOG PS and visceral disease.

	*ALK* >4 copies		P-value
	No	Yes	
**ECOG PS**			0.55
0	18	4	
1, 2	35	13	
**Visceral disease**			0.16
No	34	7	
Yes	19	10	

**Table 4:** Comparison of OS between ≥5F patients and 2F+3−4F.

	N	Death	Median OS	Hazard ratio	P-value	Adjusted hazard ratio	P-value
**AKL >4 copies**					0.80		0.38
**2F+3−4F**	53	34	12	1.1 (0.55, 2.16)		1.36 (0.60, 2.72)	
**≥5F**	17	11	16	1 (reference)		1 (reference)	

Comparison of *ALK* gene copy number gains to clinical and pathological features for the 70 patients are summarized in **Table 1**. There were no differences between *ALK* gene copy number gains and clinical features in all the subgroups (2F, 3–4F and ≥5F). OS rates for patients with 2F+3−4F and >5F were 12 and 16 months respectively. There was no statistically significant difference between these groups (**Figure 3**).

### *ALK* protein expression by immunohistochemistry

To further characterize whether *ALK* protein expression was affected, immunohistochemistry analysis of all FFPE samples was performed using the Cell Signaling antibody. Immunohistochemistry staining was negative in the tumor with *ALK* FISH positive test. Similarly, among tumors with *ALK* gene copy gain or amplification, *ALK* protein expression was not detected. None of the tumors classified as *ALK* negative by FISH showed *ALK* protein expression by immunohistochemistry.

### High-throughput mutational analysis using Oncomap

To have more accurate information on genetic alteration in these UC samples, mass spectrometry mutation analysis was also performed for all samples. Ninety-six samples were submitted for OncoMap: 87/96 (91%) passed all quality control steps. 79% (69/87) passing samples harbored candidate mutations. In total, 150 candidate mutation calls were made across 47 genes. Overall, 39% (58/150) of candidate mutations in passing samples were conservative and 61% (92/150) were aggressive. No mutations in *ALK* were found using this platform. *ALK* P496L candidate mutation was found in one of the sample but was not confirmed with HME.

### Next-generation sequencing of *ALK* gene

Since FISH technique gives no information of the specific sequence and the exact size of the deleted fragment in *ALK*, directed analysis of *ALK* gene was performed by next generation sequencing (Illumina). Analysis of the region containing P496 only showed base changes at rates below 1%, reflecting the expected sequencing error rate. Thus, only the wild-type sequence for the position P496 was detected and no mutations on *ALK* were detected by this technique.

We then extended the search space to the centromere with the intention to explore potential deletions according to FISH results. In the new analysis performed on the FISH positive patient, one read of a pair should match within the ALK locus, 29.37 Mb - 32 Mb, and one read should match at some place towards the centromere (>10 kb up to position 93.3 Mb). However, at the coverage level used no deletions could be confirmed with this approach.

## Discussion

In the present study we interrogate whether the *ALK* genomic alterations are of potential clinical relevance in patients with UC. Our study shows that *ALK* amplification and copy number gain but not fusions and translocations occurs in UC but is not associated with poor outcome in our patients with already bad prognosis.

*ALK* gene is located in 2p23 and encodes a transmembrane tyrosine kinase receptor involved in the development of nervous system during embryogenesis [20], [21]. *ALK* gene was first shown to have a role in cancer as part of the fusion gene nucleophosmin (NPM)-*ALK* in anaplastic large cell lymphomas(9, 10). Preclinical studies show that tumors with aberrant activation of *ALK* tyrosine kinase are oncogene addicted to *ALK* intracellular signaling, and inhibition of the kinase by specific *ALK* targeting drugs results in tumor growth arrest and cell death (25).

The best well studied genomic alteration is the translocation seen in NSCLC patients. The majority of *ALK* rearrangements come from an interstitial deletion and inversion in chromosome 2p resulting in EML4–*ALK* fusion gene product [22]–[27].Although translocation is the most commonly identified mechanism for *ALK* activation, amplification and mutation have also been shown to act as oncogenic events [28]–[30]. The role of amplification and of copy number gain, as well as the role of deletion found in tumors like RMS remains to be determined [27], [31]–[34].

The finding that several tumor types have been identified that have *ALK* as an oncogenic driver regardless of their cell of origin has prompted the creation of the term “*ALK*omas” implying a “beyond organ” concept classification assuming consequently responses to *ALK* inhibitors such as crizotinib [10], [35]. Based on that, exploration of this concept is worthwhile in UC even if the frequency happens to be low.

In our cohort, aCGH-A found only some minor focal events in 3/70 specimens harboring non-significant alterations in *ALK* gene locus region. Since copy number gain has been recently associated with poor prognosis in several tumors like RMS, RCC and colorectal cancer (CRC), FISH analysis to assess the impact of copy number variations of *ALK* in our cohort was performed. In our patients, polysomy was frequently found in 90% of the cases [15], [34]. The biological relevance of such finding is uncertain but could reflect genomic instability. The OS for patients with (2F+3−4F) vs. >5F was found to be 12 and 16 months respectively, however did not reach statistical significance (**Figure 1**). Likewise, there were no differences between *ALK* gene copy number gains and clinical features in all the different subgroups (2F, 3–4F and ≥5F). A plausible explanation for this lack of a significant difference between these groups is that it could be related to the natural history and the aggressive phenotype of our analysis cohort (metastatic disease requiring chemotherapy) with other genetic abnormalities beyond *ALK* gene copy number having a greater functional role in oncogenesis. Similarly, arm level ALK gene copy number gain as observed in this analysis may be unrelated to the driver oncogenic events.

Generally, patients with *ALK* copy gain have not shown to have detectable *ALK* protein expression as assessed by IHC except for a recent publication by van Gaal and colleagues [27], [29], [34], [36]. In our series, no patient with gene copy gain or amplification tested positive by IHC. This is similar to that observed in CRC where increased *ALK* gene copy number did not translate to increased *ALK* protein expression [37]. However, this is not the case for patients being categorized as FISH positive, where this positivity strongly correlates with IHC. Of note, in lung cancer, a positive *ALK* FISH test and *ALK* IHC have been proposed as screening tools to detect *ALK* alterations being considered sufficiently sensitive to indicate treatment with crizotinib [37]. Moreover, in NSCLC, abnormal FISH signal patterns have varied from a single split signal to more complex signal patterns, such as deletions of the green 5′ end of the *ALK* probe, gain of the split or 5′*ALK* signal or both. These variant *ALK* FISH signals usually, but not always, represent an *ALK* translocation and therefore the finding of a loss of the 5′*ALK* signal has been considered to be a presumptive evidence of an *ALK* gene rearrangement [37].

In our series, the patient with a FISH positive result had a variant signal pattern that did not correlate with *ALK* protein expression as assessed by IHC. The case was interpreted as having a deletion in the *ALK* region due to loss of the green 5′ end of the *ALK* signal, after excluding the possibility it could be related to alternative translocation partners [Kinesin family 5B (*KIF5B*) and TRK-fused gene (*TFG*)]. In our patient we did not test for the rearrangement of other fusion partners to *ALK* such as C2orf44, *KIF*5B, *NPM*1, *VCL*, *TFG*, *RET*, *ROS*, and *VCL* [38]–[43]. These genes have all been shown to be partners of *ALK* in lung cancer [44].

Finally, *ALK* Mutations have been described in 10.4% of neuroblastoma samples but not in other pediatric tumors like RMS, Ewing sarcoma, or DSRCT and only occasionally in other solid tumors like CRC [45], [46]. In lung cancers, *ALK* mutations appear to develop during clinical treatment with crizotinib and their generation probably renders EML4-*ALK* resistant not only to crizotinib but also to other *ALK* inhibitors [47]. In our series, no *ALK* P496L mutation was observed. In our study the limitations of the platform used limits our conclusions of the mutation analysis. The absence or very low percentage of activating mutation of *ALK* described in the majority of adult solid tumors tested support our analysis that these alterations are not relevant events in UC. Unfortunately, the suspected deletion in the *ALK* region was not confirmed with the sequencing approach used. Discordantly, mapping read pairs suggesting deletions resolved into correctly mapping read pairs that were in agreement with the insert size of the library when a single mismatch between read and reference genome was tolerated. Thus, these pairs do not support deletions at the *ALK* locus. The average read coverage across the *ALK* region was 5× and if only a small proportion of cells contained a deletion, we would not have been able to detect it. Because we suspect the deletion was close to the centromere, we might have missed it and might not have been able to confirm it by next generation sequencing.

To summarize, the increasing evidence that *ALK* alterations are seen in tumors from different origins highlights the concept of stratifying tumors according to oncogenic genotypes as opposed to tissue type when considering treatment strategies. The finding of the absence of *ALK* rearrangement together with no activating mutation in *ALK* suggests that these alterations might not be pathogenic events in UC. The utility of testing ALK inhibitors in UC is not supported by this data, although in the absence of effective alternative agents testing ALK inhibitors may still be warranted.

In conclusion, *ALK* genomic alterations are rare and probably without prognostic implications in UC. The potential for testing *ALK* inhibitors in patients with deletions and copy number changes UC merits further investigation in a larger expanded cohort of UCs, but might be restricted to the infrequent finding of a FISH positive patient.

## Supporting Information

Methods S1 **Supplementary Methods.**
(DOCX)
