# Supplementary material for: Identification of ALK Gene Alterations in Urothelial Carcinoma
Source: PLoS One. 2014 Aug 1;9(8):e103325. doi: 10.1371/journal.pone.0103325 (PMC4118868; doi:10.1371/journal.pone.0103325)
Supplement: Methods S1 — Supplementary Methods. (DOCX) [file pone.0103325.s001.docx]

**Supplementary Information**

**Methods S1**

**Array Comparative Genomic Hibridization (aCGH)**

DNA from primary tumors was hybridized to Agilent Oligonucleotide human genome 180k CGH-Array. Normal male 46XY genomic DNA was obtained from Promega (Madison, WI) as a control. Genomic DNA was extracted from specimens using QIAamp DNA FFPE Tissue Kit (Qiagen, Valencia, CA). The Genomic DNA ULS labeling kit for FFPE Samples (Agilent) was used to chemically label 500ng of genomic DNA with either ULS-Cy5 (tumor) or ULS-Cy3 dye (normal/reference DNA) according to the manufacturer's protocol (Agilent Technologies, Inc., Palo Alto, CA). Samples were hybridized to the Agilent SurePrint G3 Human CGH+SNP Microarray 4x180K. Each slide contains 4 identical arrays consisting of approximately 170,334 in situ synthesized 60-mer oligonucleotide probes that span coding and non-coding sequences with an average spatial resolution of 13 kb. The sample was applied to the array using an Agilent microarray hybridization chamber and hybridization was carried out for 40 hours at 65°C in a Robbins Scientific rotating oven at 20 rpm. The arrays were then disassembled according to the manufacturer's protocol. Slides were dried and scanned using an Agilent DNA microarray scanner. CGH Analytics software version 3.4 (Agilent Technologies, CA) was used to analyze the CGH-A data.

Normalized copy number data was first segmented using GLAD[[14](#_ENREF_14)]with default parameters available in GenePattern version 3.3.3. The Genomic Identification of Significant Targets in Cancer (GISTIC) softwar[e](#h.1ci93xb) (v2.0.12)[[15](#_ENREF_15)] was then used to identify regions of the genome that were significantly altered across multiple samples. Imbalances of >750 kb and1<10 Mb in sequence length were classified as focal, and imbalances > 10 Mb were categorized as segmental. A contiguous change of imbalance level within a chromosome was considered to be indicative of an unbalanced translocation event.

**Fluorescence *in situ* hybridization (FISH)**

TMAs were hybridized with a FISH probe targeting *ALK* gene and were scored for copy number gain or amplification by two cytogeneticists. To assess the genetic status of *ALK*, we used dual-color break-apart probes flanking the *ALK* gene (Abbott Molecular Inc, Des Plaines IL, USA). This probe cocktail contains two differentially labeled probes on opposite sides of the breakpoint of the *ALK* gene at 2p23. A probe (approximately 250 kb), was labeled with Spectrum Orange (3’*ALK*) on the telomeric side of the *ALK* breakpoint. The centromeric probe (approximately 300 kb) was labeled with SpectrumGreen (5’*ALK*). In a serial TMA tissue section, FISH with CEP2 (a centromeric alpha-satellite specific for chromosome 2 was performed (Abbott Molecular Inc.) to exclude polysomy.

In one case that had positive FISH pattern for *ALK*, FISH for the echinoderm microtubule-associated protein-like 4 (*EML4)* (2p23), Kinesin family 5B (*KIF5B)* (10p11.22), and TRK-fused gene (*TFG)* (3q12.2) was performed. These genes have all been shown to be partners of *ALK* in lung cancer[[16](#_ENREF_16)]. Break-apart probes were designed using bacterial artificial chromosome (BAC) clones selected from the CHORI BAC/PAC resource ([*http://bacpac.chori.org*](http://bacpac.chori.org)). The following probes were created: centromeric *EML4*; pooled RP11-804P20 and RP11-413N9; telomeric *EML4*: pooled RP11-798D22 and RP11-34L01; centromeric *KIF5B*; pooled RP11-633K11; and RP11-281A19 in spectrum Red, telomeric *KIF5B* pooled RP11-460H18 and RP11-166N17; centromeric *TFG*: pooled RP11-320C17 and RP11-423M9, telomeric *TFG*: pooled RP11-49H3 and RP11-168G7. Results were analyzed in a fluorescent microscope (Olympus, BX51) using the Cytovision software (Applied Imaging, Santa Clara, CA), and scoring a minimum of 50 nuclei.

Normal fusion *ALK* signals presented as an overlapping orange and green (yellowish) signals. This probe was considered rearranged if >15% of tumor cells showed split green and orange signals (at least by two times the signal diameter), and atypically rearranged when a single orange signal was seen. Gains (denoted as F for fusion signal) were defined as mean copy number of 3-6 fusion (3-6F) signals in more than 10% of cells. For example, a tumor was denoted as 2–4F that had 4F signals in 10 of 200 nuclei (5%), 3F signals in 30 of 200 nuclei (15%), and 2F signals in the remaining nuclei. Alternatively, a tumor with 2 of 200 nuclei (1%) showing 8F signals and the remaining nuclei having 3F signals was given a score of 3F. The 10% value was selected to reduce the fusion signal artifact due to nuclear overlap, while maintaining sensitivity for the presence of additional *ALK* genes in a small subset of tumor cells due to tumor heterogeneity. Amplification of the *ALK* gene was defined as *ALK*/CEP2 ratio greater than 2.

**Immunohistochemistry**

Four micrometers serial sections of FFPE biopsies were used for the evaluation of *ALK*, using the *ALK*-1 antibody from Cell Signaling. Diaminobenzidine was used as the cromogen to identify positive results. For *ALK* expression scoring, the percent of cells was determined within each staining intensity category 0-4+ and a hybrid score (H-score) was calculated by the formula: *% cells of 0 intensity+(%cells of 1 intensity*1) +(%cells of 2 intensity*2)+ +(%cells of 3 intensity*3)+(%cells of 4 intensity*4)*. An EML4-*ALK*-translocated NSCLC case served as a positive control for *ALK*. Normal tissue was used as negative control.

**Mass spectrometry mutation analysis**

For the mass spectrometry mutation analysis, the OncoMap 3 Core and Extended analysis for the OncoMap Profiling of Bladder Cancer was used looking for *ALK* P496L mutation. The complete mutation profiling algorithm involved mass spectrometric genotyping followed by both automated calling and manual review to generate a list of candidate mutations. The analysis was done using DNA derived from FFPE clinical material. Genomic DNA was quantified using Quant-iT PicoGreen dsDNA Assay Kit (Invitrogen) per manufacturer's protocol. 250ng sample of DNA was used for mutation analysis using OncoMap mass spectrometric genotyping based on the Sequenom MassARRAY^®^ technology (Sequenom Inc, San Diego, CA) and performed as previously described with some modifications[[17](#_ENREF_17)]. Probes were designed that enabled mutation detection. Mass spectrometric genotyping using iPLEX chemistries were performed (Sequenom Inc., San Diego, CA) by extending the probes with one base in the presence of chain-terminating di-deoxynucleotides that generate allele-specific DNA products. The extension products were spotted onto a specially designed chip and analyzed by matrix-assisted laser desorption/ionization-time of flight (MALDI-TOF) mass spectrometry to determine the mutation status based on the difference in mass of the mutant and wild type (WT) base.

An automated mutation-calling algorithm was performed to identify the candidate mutation *ALK* P496L. Putative mutations were further filtered by a manual review and selected for validation using multi-base homogenous Mass-Extend (hME) chemistry. Only if the mutations found in iPLEX were confirmed by hME they were considered validated mutation.

The sensitivity and specificity of OncoMap is 93.8% and 100% in fresh frozen tissue and 89.3% and 99.4% in FFPE-derived DNA[[17](#_ENREF_17)]. This sensitivity greatly exceeds that of Sanger sequencing, which remains the gold standard for many genetic diagnostic approaches.

**Next-generation sequencing**

For the detection of a mutation at *ALK*-P496, 15 different amplicon were generated spanning the mutation site, sequencing libraries were prepared by ligation of Illumina adapters to PCR fragments according to published procedures. The ends of DNA fragments were made blunt by end-repair with T4 DNA polymerase and Klenow fragment. Thereafter, 3’-adenylation was performed by incubation with dATP and exo-Klenow fragment. Double-stranded adapters were ligated to the DNA using rapid T4 DNA ligase. On a 2% agarose gel, fragments in the size range of interest were excised with a sterile scalpel and recovered from the gel. Adapter-ligated fragments were enriched, and adapters were extended in an 18-cycle PCR reaction. Finally, the quality of library was confirmed on the Agilent 2100 Bioanalyzer. In a concentration of 8pM, the library was loaded onto a v4 single read flowcell (IIllumina). Clusters were prepared on the Illumina cluster station. The flowcell was loaded onto the Illumina Genome Analyzer IIx, and 36 sequencing cycles were performed using Illumina TruSeq sequencing chemistry. Base calling was performed using Illumina RTA version 1.7.0 (within SCS2.9).

All amplicon were sequenced in a single lane, and a total of 56 million raw reads were obtained. Specific reads were selected that contained a perfect match to either forward or reverse PCR primers that were used to amplify an amplicon from the *ALK* gene. Thereafter, read filtering was performed. The filtering included chastity filtering, removal of reads with uncalled bases, and removal of reads with low quality bases. In total, about 10% of the reads that had a perfect match to a PCR primer were removed in this filtering step. The final data set for the *ALK* amplicon contained 1.1 million sequence reads with a perfect match to the *ALK* forward primer at read start, and 0.9 million reads with a perfect match to the *ALK* reverse primer at read start. Reads were clustered and the numbers of the most frequent changes in comparison to the reference sequence were counted.

To search for a deletion that affects the *ALK* locus, whole genome shotgun paired end reads from a library with an insert size of 355 bases were generated on an Illumina HiSeq 2000 sequencer. One lane was sequenced resulting in 187,184,122 raw read pairs.

Read pairs were mapped to the unmasked human genome hg19 (GRCh37/hg19) downloaded from NCBI. Mapping was performed with a Burrows-Wheeler Alignment (BWA) tool allowing for up to 3 edits[[18](#_ENREF_18)]. Read pairs were discarded if they had no read placed uniquely between the *ALK* locus and the centromeric region at chromosome 2 (chr2 29.37 Mb – 93.3 Mb). We retained 3,595,671 read pairs. The average read coverage in this region was 5x. In order to detect large deletions, which affect the *ALK* locus, we further discarded read pairs that did not match with at least one read within the *ALK* locus (chr2 29.37 Mb - 32. Mb) and those pairs that mapped at a distance of less than10,000 base pairs. The remaining read pairs were clustered based on their mapping coordinates. Two or more read pairs were clustered if upstream mapping reads and the downstream mapping reads aligned within a distance of 1000 bases, respectively. Clusters were discarded if they contained no read pair with uniquely placed reads or if all reads overlapped. After applying all of these filtering steps, two clusters remained representing two potential, non-overlapping deletions of 3.6 Mb and 765 kb, respectively. However, neither of them can be considered as bona fide deletions because both clusters had alternate mapping locations which were in agreement with the expected distance between paired-end reads.
